# Professor Finsen

**Published:** 1904-10-01

**Authors:** 


### Professor Finsen.

The death of Professor Finsen may appropriately be described as the death of one who has made substantial contributions to the relief of mankind. This is not the time to endeavour to measure the full value of his work or to define its exact position in the series of triumphs by which the forces of nature have been made to render service to human welfare. Yet it cannot be doubted that history will award him the recognition reserved for the gifted few who penetrate some secret of nature to which all others are blind and initiate an impulse that leads to large and beneficial achievement. Pathetic as are many of the aspects of Finsen's life and death, he at least had this good fortune, that his ability and insight were united to a practical application which received so wide and ready a recognition that to-day many who never saw his face, and to whom he was but the shadow of a great name, will mourn his loss as that of their greatest friend and benefactor. With the expert's appreciation of his scientific ideas, and of the manner in which he cultivated and developed them, will be mingled tributes of personal gratitude and regret from many lands. It may be that the advance of knowledge will lead in greater or less measure to the supersession of the therapeutic method associated with his name, but it will ever remain true that through him there came to numerous men and women relief from a horrible and disfiguring disease. Hence it is inevitable that at the present moment the personal relationships of the man and his work should mainly occupy the general attention. Prominent in this respect will be the thought that a life so valuable should have had so brief a course. It is impossible to resist the reflection that its chapter of achievement contained the promise of much further service, and that the end has come all too soon. Certainly it will not be forgotten that a brave spirit was not quenched, was indeed hardly hindered, by severe and prolonged personal suffering and bodily ill-health. The sustained enthusiasm for science and for the application of knowledge to practical ends in the treatment of disease carried the man over obstacles which could only have been overcome by a generous and noble unselfishness. Hence humanity not less than science mourns the death of one of its truly heroic sons. Regarding the nature and method of Finsen's work it is worthy of note that the institution of his therapeutic scheme was not the outcome of some chance thought or happy inspiration. On the contrary, it emerged from a careful and thoughtful observation of nature, it grew to maturity under the effort of prolonged labour, and it was tested and studied in the true scientific spirit. Perchance it may be shown that others before his day had some appreciation of the value of light as a therapeutic agent but it was for Finsen to grasp the full significance of this suggestion, to apply it on scientific lines, and to extend it by a practical method. It was in this way he showed the initiative capacity and compelling quality of true genius and it is here that the promise of the permanence of his influence may be found. To say in these days of scientific revolutions that all the details of his method, or even the method itself, will certainly be permanent would be a rash prophecy. It is both more wise and more satisfactory to reflect that in addition to its present practical value the work has in it the possibility of development and that it has stimulated new lines of research the beneficial applications of which are probably but in their infancy. It is to men who have the insight to see and the courage to follow new lines of progress that the larger recognitions of mankind are paid, and there is every reason to believe that such a verdict will be awarded to the work of the Danish physician. At the close of that work there is some satisfaction in reflecting that in its inception and development it commanded the sympathy and encouragement of those who were able to secure for it the needed opportunity, and the people of this country have special reason for remembering with gratitude and pride some of the influences which helped its extension. The work remains in the service of the race far beyond the place and nation of the man who achieved it. For him there is the not unworthy reward that he "shall be well spoken of upon earth by those that are just, and of the family of virtue."

